# CPEB4 Inhibit Cell Proliferation via Upregulating p21 mRNA Stability in Renal Cell Carcinoma

**DOI:** 10.3389/fcell.2021.687253

**Published:** 2021-12-16

**Authors:** Jiehui Di, Hui Wang, Zhongjun Zhao, Guang Zhao, Xiaobing Qin, Zhengxiang Han, Yong Liu

**Affiliations:** ^1^ Cancer Institute, Xuzhou Medical University, Xuzhou, China; ^2^ Center of Clinical Oncology, Affiliated Hospital of Xuzhou Medical University, Xuzhou, China; ^3^ Jiangsu Center for the Collaboration and Innovation of Cancer Biotherapy, Cancer Institute, Xuzhou Medical University, Xuzhou, China; ^4^ Department of Oncology, The Affiliated Hospital of Xuzhou Medical University, Xuzhou, China

**Keywords:** CPEB4, proliferation, p21, renal cell carcinoma, mRNA stability

## Abstract

Cytoplasmic polyadenylation element-binding protein 4 (CPEB4) has been reported to be dysregulated in a variety of cancers and seems to play paradoxical roles in different cancers. However, the functional roles of CPEB4 in Renal cell carcinoma (RCC) are still unclear. This study aims to explore the role and underlying mechanism of CPEB4 in RCC. We found that the relative expression level of CPEB4 is down-regulated in RCC tissues and cell lines, and the low CPEB4 expression is correlated with short overall and disease-free survival of RCC patients. CPEB4 significantly inhibits RCC tumor growth both *in vivo* and *in vitro*. CPEB4 exerts an anti-tumor effect by increasing p21 mRNA stability and inducing G1 cell cycle arrest in RCC. Our data revealed that CPEB4 is a tumor suppressor gene that restrains cell cycle progression upstream of p21 in RCC. These findings revealed that CPEB4 may become a promising predictive biomarker for prognosis in patients with RCC.

## Introduction

Renal cell carcinoma (RCC) is the most lethal malignancies of the urinary system, and the incidence rate of RCC is continuously increasing in recent years (Yang and Chen, 2020). Although the diagnosis and treatment of RCCs have improved substantially these years, but the treatment response varies and most patients continue to progress (Singh, 2021). The pathogenesis of RCC involves the alteration of varieties of intracellular gene expression (Delahunt et al., 2021). Therefore, the research for strengthening the molecular mechanism of renal cancer development and identifying novel potential biomarkers is still largely necessary to the diagnosis and treatment of RCC.

Many processes of tumor development are due to dysregulated gene expression (Hanahan and Weinberg, 2011). RNA binding proteins (RBPs) are involved in almost all steps of post-transcriptional regulation, whose abnormal expression are closely related to the occurrence and development of many cancers. Cytoplasmic polyadenylation element binding protein 4 (CPEB4) is a sequence-specific RBP that belongs to the CPEB family (Kurihara et al., 2003). CPEBs binds the cytoplasmic polyadenylation element (CPE; with a consensus sequence of UUUUUAU) in the 3′-untranslated regions (3′-UTR) of target mRNAs to regulate mRNA stability and translation by promoting cytoplasmic polyadenylation (Ivshina et al., 2014), which mediate many biological processes including germ-cell development, cell division, cellular senescence, synaptic plasticity, and learning and memory (Richter, 2007; Chen et al., 2016).

CPEB4 was first identified to be upregulated and acted as an oncogene in pancreatic cancer (Ortiz-Zapater et al., 2011). After that, CPEB4 expression was found aberrantly expressed in several cancers. CPEB4 is upregulated and correlated to poor prognosis in patients with glioma and gastric cancer (Hu et al., 2015; Cao et al., 2018). However, CPEB4 is downregulated in hepatocellular carcinoma and non-small cell lung cancer (Tian et al., 2012; Huang et al., 2015). CPEB4 seems to play paradoxical roles in different cancers, and the expression and function of CPEB4 in RCC remains unknown.

One of the main forces that drives cell transformation is the loss of proper control of cell cycle. The cyclin-dependent kinase inhibitor CDKN1A (p21) acts as a well-known tumor suppressor in many types of cancers, because p21 is one of the most important target of p53 and functioned as cell-cycle checking point to inhibit cancer cell over proliferation (Abbas and Dutta, 2009). p21 expression and protein activities are modified by multiple mechanisms both at the transcriptional and post-translational levels (Hsu et al., 2007; Lu and Hunter, 2010). Because of the important role of p21 in cancer development, it is vital to explore the mechanisms underlying the dysregulation of p21 in RCC.

In this study, CPEB4 was found to be downregulated in RCC tissues and inhibited RCC cell proliferation both *in vivo* and *in vitro*. Mechanistically, CPEB4 upregulates the expression of p21 by increasing p21 mRNA stability, and further induces G1 cell cycle arrest. CPEB4 modulates RCC cell proliferation by inhibiting cell cycle progression partially through increasing p21 expression. Overall, our findings suggests that CPEB4 may serve as a new anticancer therapeutic target in RCC treatment.

## Materials and Methods

### Clinical Specimens

Renal tumors and adjacent tissues were obtained from the Affiliated Hospital of Xuzhou Medical University (Xuzhou, China). This research was approved by the Ethical Review Committee of this hospital. The specimens were collected and stored in liquid nitrogen immediately after the surgery. Total protein was extracted and subjected to WB analysis.

### Cell Culture

Four human RCC cell lines (ACHN, OSRC2, 786O and Ketr-3) and one normal renal tubular epithelial cell line (HK2) were obtained from the Cell Bank of the Shanghai Institutes of Biological Sciences, Chinese Academy of Sciences. HK2, ACHN and Ketr-3 cells were cultured in DMEM high glucose medium, and the 786O and OSRC2 were cultured in RMPI 1640 medium (Sigma, United States) with 10% fetal bovine serum in a 37°C, 5% CO_2_ incubator (Thermo Scientific, United States).

### Transfection

To suppress CPEB4 expression with siRNA, the cells were cultured to 30–50% confluence, and then transfected for 48 h with siRNAs that target CPEB4 or with a nonspecific control (NC). All transfections were performed using siLentFect (Bio-Rad) according to the manufacturer’s instructions. siRNAs specifically targeting CPEB4 or nonspecific control (NC) were synthesized by GenePharm, and their sequences were listed in Supplementary Table S1. The CPEB4 sequence (CPEB4A) was subcloned into the lentiviral vector pCD513B. The virus was packaged in HEK-293T cells and then used to infect 786O and ACHN cells. For stable knockdown of CPEB4, 786O and ACHN cells were separately transduced with each of two CPEB4 shRNAs cloned in the pLKO.1 vector, and selected in the presence of puromycin.

### RT-qPCR

Total RNA was extracted from cultured cells using TRIzol reagent according to the manufacturer’s instructions (Invitrogen). cDNA was synthesized using a PrimeScript reverse transcription (RT) reagent kit (TaKaRa) in the presence of gDNA Eraser. Quantitative real-time PCR was carried out in a ViiA 7 real-time PCR system (Applied Biosystems) using a SYBR Premix Ex Taq II kit (Takara). The primer sequences used in RT-PCR were listed in Supplementary Table S1.

### Western-Blot

Cells or tissue specimens were lysed in an NP-40-containing buffer. Equal amounts of protein were separated by SDS-PAGE. After incubation with the indicated antibodies, the immune complexes on the membrane were detected by an ECL kit (Thermo Scientific, #32106). The following antibodies were acquired from commercial sources: rabbit anti-CPEB4 (Proteintech, #25342-1-AP) and rabbit anti-p21 (Santa cruz, #Sc-6246).

### Immunofluorescence Staining

Cells grown on cover slips in a 24-well plate were fixed with 4% paraformaldehyde for 20 min and then treated with 0.1% Triton X-100 solution on ice for 4 min. Samples were then blocked with 3% BSA for 1 h followed by antibody incubation at 4°C overnight. Then, the cells were washed 3 × 5 min in PBS and incubated with the fluorescently labeled secondary antibodies for 1 h. DAPI was used to stain the nuclei for 3 min. The slides were mounted with 90% glycerol, and the images were captured by a Zeiss Axio Observer confocal microscope.

### Cell Proliferation and Colony Formation Assays

Cell proliferation assays were performed using a cell counting kit-8 (CCK-8) from Beyotime Institute of Biotechnology (Nanjing, China). For the colony formation assay, 200 cells were seeded into 6-well plates. After incubation for 14 days, the plates were washed with PBS three times, and the colonies were fixed with 4% paraformaldehyde solution and stained with 0.5% crystal violet. The number of the colonies was counted by ImageJ software. The experiments were independently repeated three times.

### Xenograft Assay

To assess the influence of CPEB4 on tumorigenesis *in vivo*, 6-week-old nude female BALB/c mice were purchased from Beijing HFK Bioscience. The mice were subcutaneously injected with the indicated ACHN and 786O cells (5×10^6^ cells in 100 µl of serum-free medium containing 0.25 v/v Matrigel) in each flank. The tumor volumes were measured every 3–4 days and calculated as length × width^2^ × 0.5. The mice were sacrificed, and the tumors were carefully removed, imaged, and weighed at the end of the experiment.

### Cell Cycle Analysis

Approximately 5 × 10^5^ RCC cells were plated in a 6-well plate and maintained in medium containing no serum for 48 h, after which they were released into complete growth medium for 24 h. After release the cells were harvested and fixed using 75% pre-cooled ethanol at 4°C overnight. After being washed three times with phosphate buffered saline (PBS), cells were stained with propidium iodide (400 μg/ml) and RNase A (20 mg/ml) at room temperature for 30min, samples were then analyzed using a FACSCanto flow cytometer (BD Biosciences, San Jose, CA), data on cell cycle distribution were analyzed using ModFit LT 3.0 software.

### RIP

Cells were collected in lysis buffer (5 mM PIPES. pH 8.0, 85 mM KCl, 0.5% NP40, 1% SDS, 10 mM EDTA, and 50 mM Tris-HCl, pH 8.1) supplemented with a protease inhibitor cocktail and an RNase inhibitor (Thermo Fisher). The cell lysates were precleaned with protein G Sepharose beads and then incubated with the indicated antibodies or IgG control on a rotator at 4°C overnight. The antibody-RNA complexes were collected. The immunoprecipitated RNA was eluted and extracted for real-time PCR analysis.

### Statistical Analysis

All data were analyzed with GraphPad Prism 5 and are presented as the mean ± S.D. Student’s t test or one-way ANOVA with Dunnett’s post hoc test was used for statistical analyses of the data when appropriate. *p < 0.05, **p < 0.01, and ***p < 0.001 were considered significant.

## Results

### CPEB4 Expression is Downregulated in RCC and Positively Associated With Overall and Disease-Free Survival of RCC Patients

**FIGURE 1:**
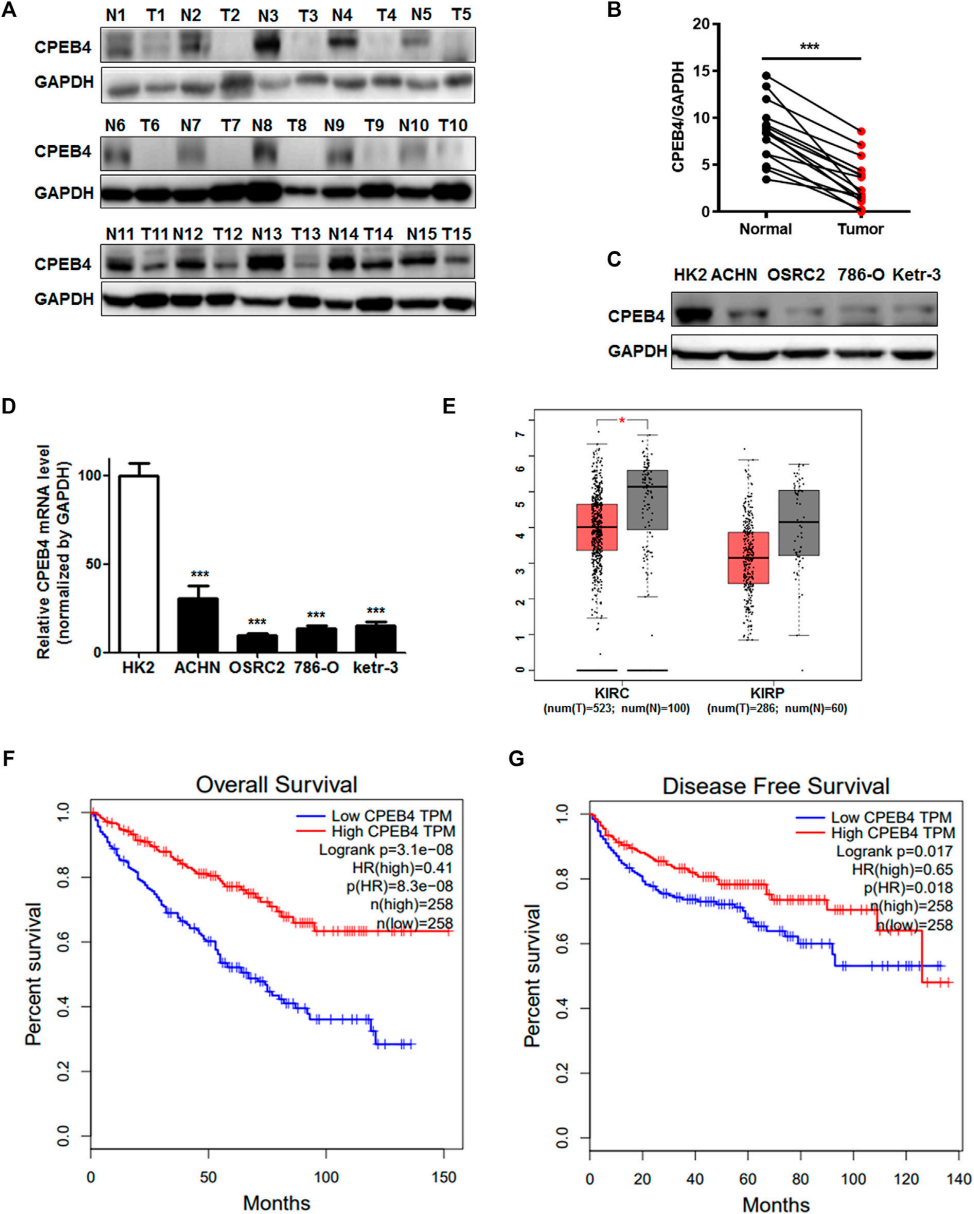
CPEB4 expression is downregulated in RCC and positively associated with overall and disease-free survival in RCC. **(A, B)** WB analysis of CPEB4 in 15 clinical RCC samples and matched adjacent normal tumor samples. **(C)** Protein level of CPEB4 in an renal tubular epithelial cell line and four RCC cell lines. **(D)** mRNA level of CPEB4 in the HK2 cell line and four RCC cell lines. **(E)** Expression of CPEB4 in TCGA database (red color is tumor tissue and gray color is normal tissue). **(F)** Overall survival rate of patients with RCC according to the mRNA expression of CPEB4, the cut off value was median expression of CPEB4 in the cohort. **(G)** Disease-free survival rate of patients with RCC according to the mRNA expression of CPEB4, the cut off value was median expression of CPEB4 in the cohort.

To determine whether CPEB4 is involved in RCC development, we first assessed CPEB4 protein expression in 15 pairs of RCC specimens and adjacent normal tissues, its expression significantly decreased in RCC tissues compared with normal tissues (Figures 1A,B). Additionally, CPEB4 expression in four different RCC cell lines was considerably downregulated compared with normal renal epithelial cell line HK2 (Figures 1C,D). Furthermore, data from the TCGA database proved that CPEB4 expression was decreased in RCC tissues (Figure 1E), and the patients who showed high CPEB4 expression corresponded with longer 5-years overall (*p* < 0.001) and disease-specific cumulative survival (*p* = 0.0018) than those with low CPEB4 expression (Figures 1F,G). These results indicated that CPEB4 might be functioned as a tumor suppressor gene in RCC progression.

### CPEB4 Inhibits RCC Cell Proliferation *in vitro* and *in vivo*

**FIGURE 2:**
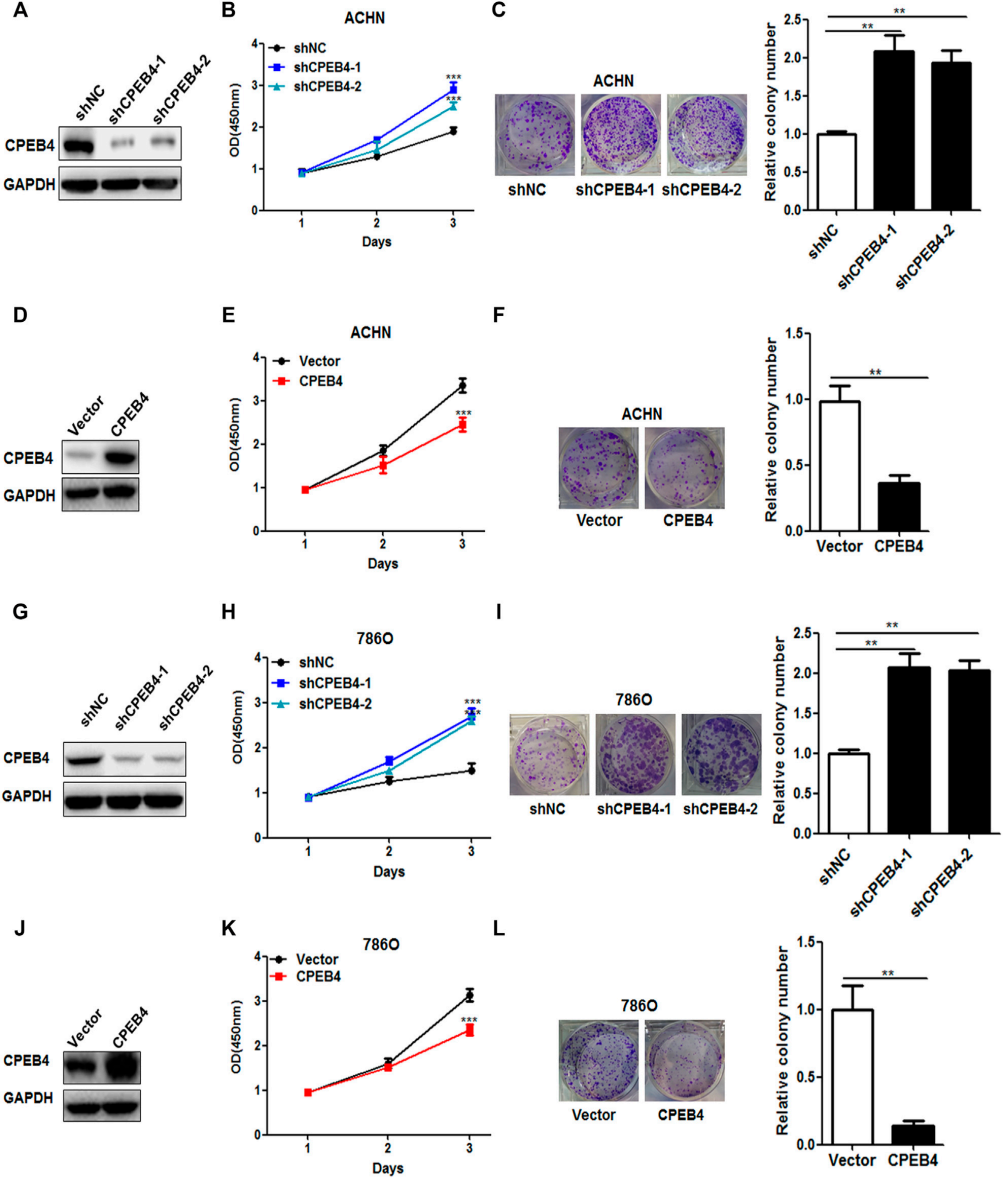
CPEB4 inhibits RCC cell proliferation *in vitro*. **(A)** The efficiency of CPEB4 knockdown in ACHN cells. **(D)** Efficiency of CPEB4 overexpression in ACHN cells. **(B,E)** Cell growth was detected by CCK-8 assays after 1, 2 and 3 days (n = 5). **(C, F)** Colony formation of ACHN cells. **(G)** Efficiency of CPEB4 knockdown in 786O cells. **(J)** The efficiency of CPEB4 overexpression in 786O cells. **(H,K)** Cell growth was detected by CCK-8 assays in 786O cells. **(I,L)** Colony formation of 786O cells.

We next examined the influence of CPEB4 on the biological behaviors of RCC cell lines. CPEB4 stable knockdown (Figures 2A,G) and overexpression (Figures 2D,J) cells were established in ACHN and 786O cell lines. The results of the CCK8 and colony formation assays showed that knockdown of CPEB4 dramatically promoted cell growth and colony formation (Figures 2B,C), and overexpression of CPEB4 suppressed cell growth and colony formation in ACHN cells (Figures 2E,F). The results were confirmed in 786O cells (Figure 2G-l), the phenotypes of the 2 cell lines are identical. According to the basal expression levels of CPEB4 compared in various RCC cell lines (Figure 1D), ACHN cells (CPEB4-high) with CPEB4 stable knockdown and 786O cells (CPEB4-low) with CPEB4 stable overexpression were used to verify the function and mechanism of CPEB4 in the follow-up experiments.

**FIGURE 3:**
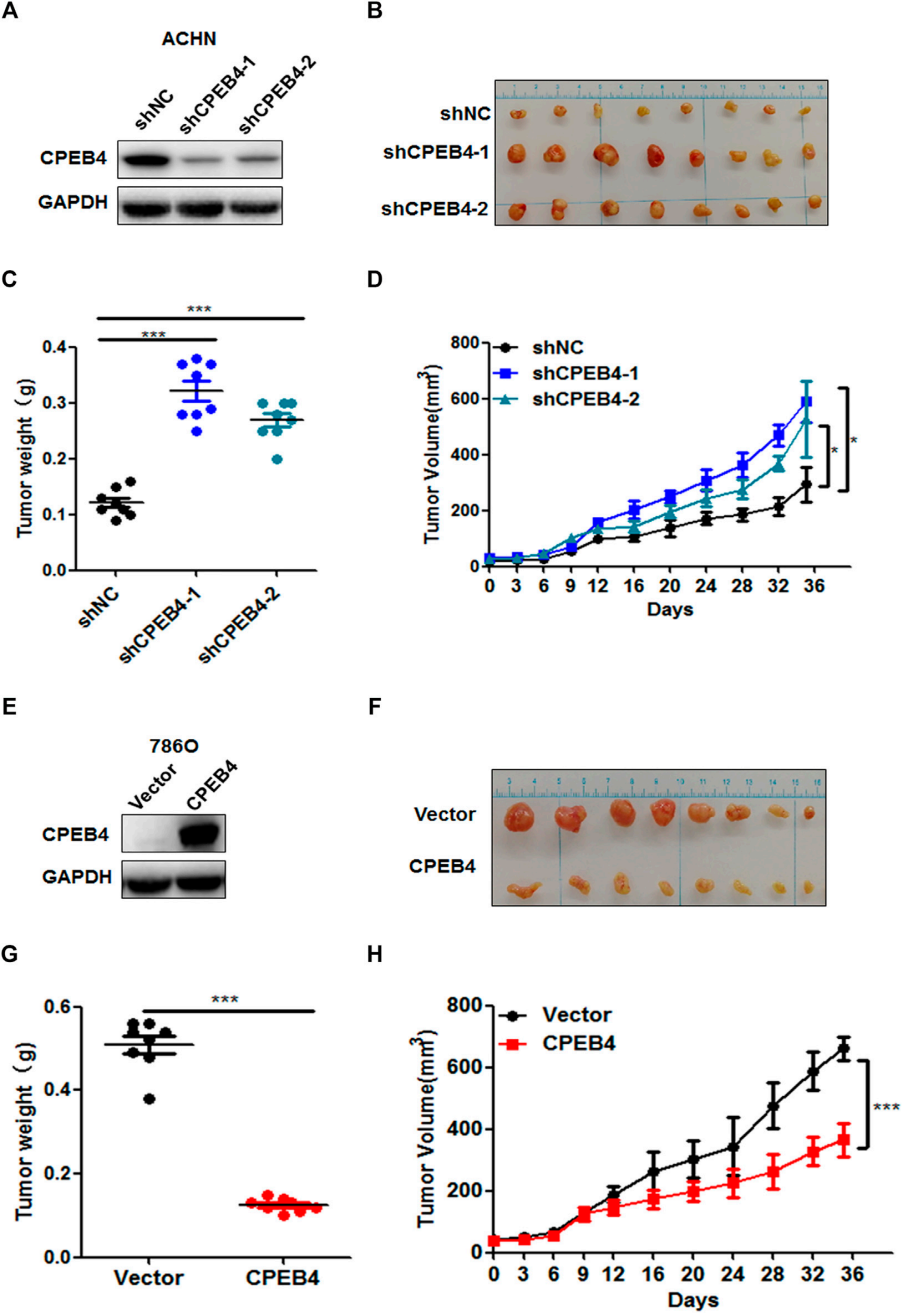
CPEB4 inhibits RCC cell proliferation *in vivo*. **(A)** The efficiency of CPEB4 knockdown in ACHN cells. **(B)** Images of the tumors of CPEB4 knockdown groups and the control group. **(C)** Tumor weights were measured after the tumors were surgically dissected. **(D)** Time course of tumor growth in mice. CPEB4 knockdown and the control cells were injected into nude mice, and tumor volumes were measured every 3 days **(E)** The efficiency of CPEB4 overexpression in 786O cells. **(F)** Images of the tumors of CPEB4 overexpression groups and the control group. **(G)** Tumor weights were measured after the tumors were surgically dissected. **(H)** Time course of tumor growth in mice. CPEB4 overexpression and the control cells were injected into nude mice, and tumor volumes were measured every 3 days.

To determine the function of CPEB4 *in vivo*, we injected CPEB4 stable knockdown and control ACHN cells (Figure 3A) that were mixed with Matrigel into BALB/c nude mice subcutaneously. After 5 weeks, we found that the tumor volume and mean tumor weight were significantly higher in the CPEB4 knockdown group than in the negative control group (Figures 3B–D). In contrast, the overexpression of CPEB4 in 786O cells (Figure 3E) markedly inhibited tumor growth *in vivo* (Figures 3F–J). Collectively, these findings suggest that CPEB4 inhibits RCC tumorigenesis both *in vitro* and *in vivo*.

### CPEB4 Induces G1 Cell Cycle Arrest

**FIGURE 4:**
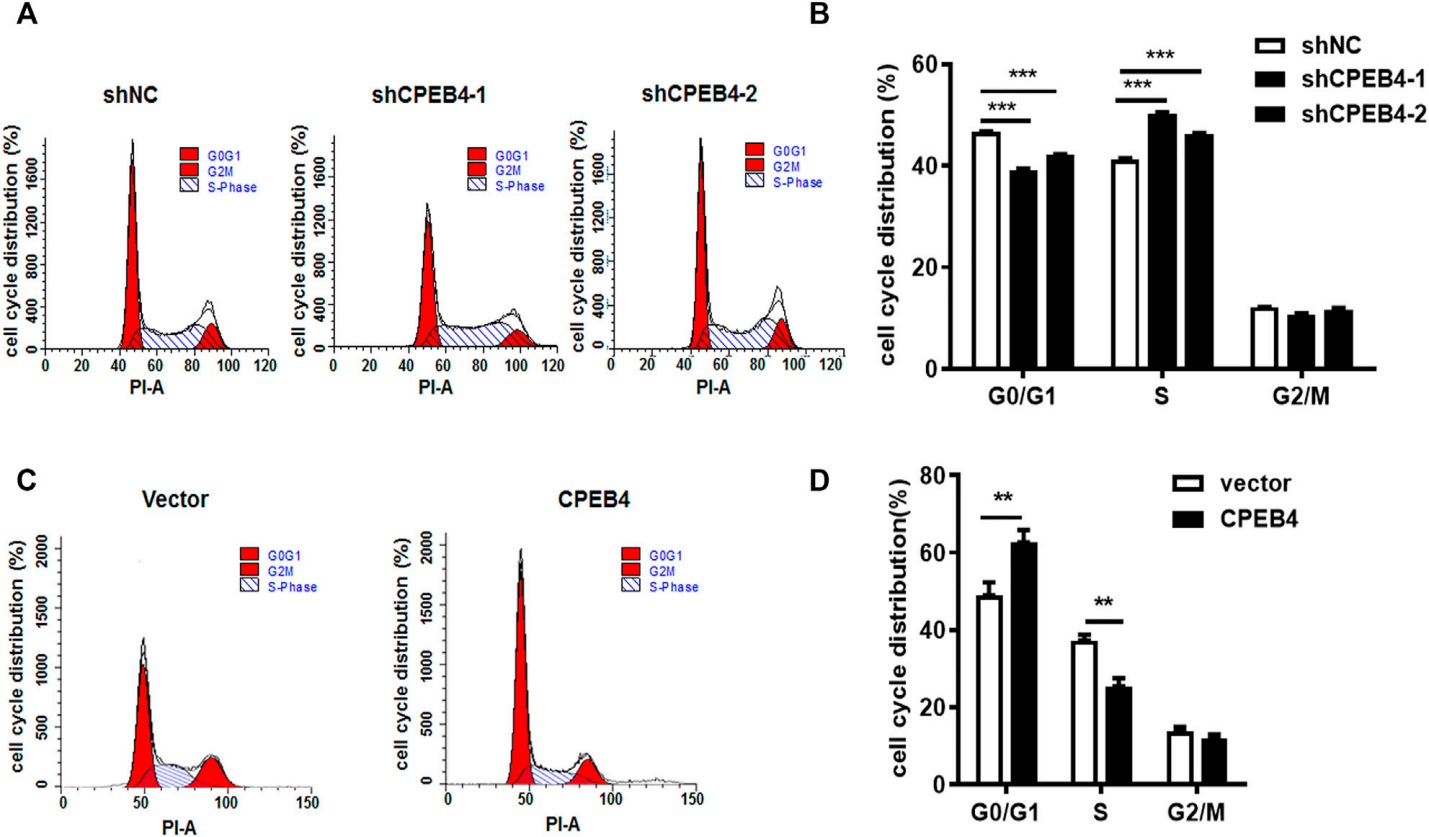
CPEB4 induces G1 cell cycle arrest. **(A,B)** Cell cycle distribution was measured by flow cytometry in ACHN cells with CPEB4 stable knockdown. **(C,D)** Cell cycle distribution was measured by flow cytometry in 786O cells with CPEB4 stable overexpression. Indicated cells were starved in medium containing no serum for 48 h, after which they were released into complete growth medium for 24 h. After release the cells were harvested and analyzed by flow cytometry. The dataset of **(A, C)** is representative example of triplicate experiments. Column graph of **(B,D)** was mean ± SD of three independent experiments.

To determine whether CPEB4 can regulate the duration of the cell cycle, we used serum starvation to synchronize RCC cells and then analysed cell-cycle progression using flow cytometry. Consistent with the CCK-8 and colony formation assay results, we found that CPEB4 knockdown promoted G1/S phase transition (Figures 4A,B). In contrast, the induction of G1 cell cycle arrest was observed when CPEB4 was overexpressed (Figures 4C,D). These results indicated that CPEB4 may inhibit RCC tumor growth by inhibiting G1/S phase transition. Recently, we have reported that CPEB2, as a p53 target, can bind to CPE elements in the p53 3′-UTR and negatively regulate p53 mRNA stability and translation. Therefore, we speculated that CPEB4 may also affect the level of p53. However, we found that CPEB4 overexpression or konckdown cannot change p53 expression, but significantly upregulate p21 expression (Supplementary Figure S1). We then detected major factors involved in cell cycle regulation, and found that only the tumor suppressor p21 mRNA expression was upregulated in 786O cells with stably transfected CPEB4 (Supplementary Figure S2).

### CPEB4 Upregulates the Expression of p21 by Increasing p21 mRNA Stability

**FIGURE 5:**
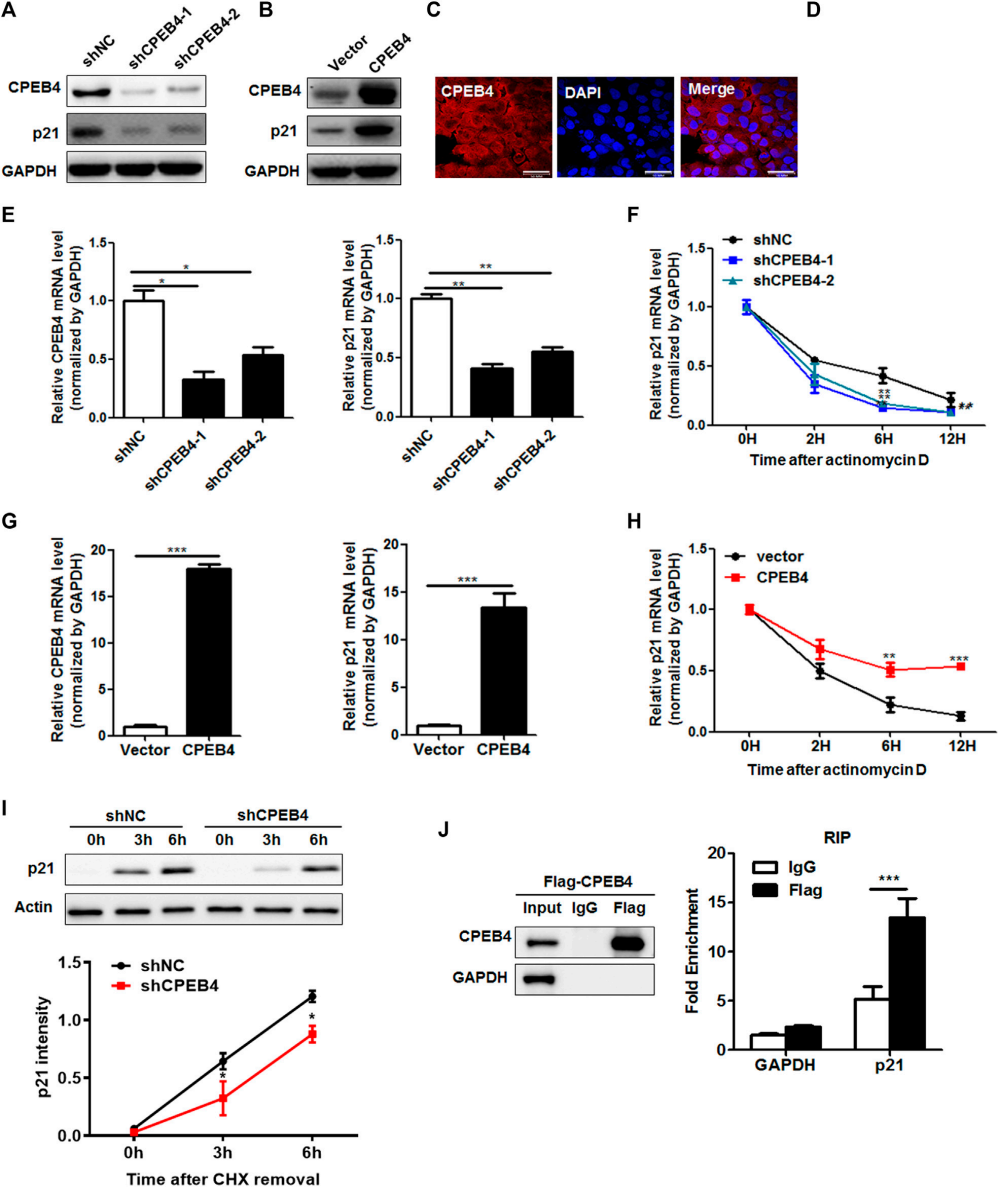
CPEB4 upregulates the expression of p21 by increasing p21 mRNA stability. **(A,B)** Western blot of p21 expression in CPEB4 knockdown or overexpression 786O cells. **(C)** The localization of Flag-tagged CPEB4 assayed by indirect immunofluorescence in CPEB4 overexpression 786O cells. **(D)** The nuclei and cytoplasm of 786O cells were isolated, and CPEB4 levels were assayed by western blot. **(E)** RT-PCR of p21 mRNA expression in CPEB4 knockdown 786O cells. **(F)** The levels of p21 transcript was measured by RT-PCR in CPEB4 knockdown 786O cells treated with actinomycin D for various times. **(G)** RT-PCR of p21 mRNA expression in CPEB4 overexpression 786O cells. **(H)** The levels of p21 transcript was measured by RT-PCR in CPEB4 overexpression 786O cells treated with actinomycin D for various times. **(I)** *de novo* synthesis of the p21 protein was analyzed by CHX removal assay. CPEB4 stable knockdown and control cells were pretreated with 100 mM cycloheximide for 12 h. After washing out cycloheximide, cells were incubated for the indicated times. p21 synthesis levels were detected by Western blot and quantified using ImageJ. **(J)** CPEB4 interaction with p21 transcript *in vivo*. The lysates of CPEB4 overexpression 786O cells were immunoprecipitated with Flag antibodies or control IgG, and RT-PCR was used to measure the transcript levels of p21 and GAPDH precipitated by Flag or IgG immunocomplexes.

Interestingly, the 3′-UTR of p21 containss CPE signal (UUUUUAU) suggesting that p21 is a potential CPEB target. Indeed, knockdown of CPEB4 significantly decreased p21 protein levels, while CPEB4 overexpression substantially enhanced p21 protein levels (Figures 5A,B). Then, we investigated whether CPEB4 regulates p21 by targeting p21 mRNA. Since mRNA is predominantly degraded in the cytoplasm, we examined the localization of ectopically expressed Flag-tagged CPEB4. The majority of CPEB4 localized in the cytoplasm (Figure 5C), which was further confirmed by the nuclear-cytoplasmic separation (Figure 5D). Consistently, CPEB4 knockdown resulted in a decrease in the p21 mRNA levels, whereas CPEB4 overexpression had an opposite effect (Figures 5E,G). Next, we tested whether the changes in the expression of p21 transcripts induced by CPEB4 are due to altered mRNA stability. For this purpose, actinomycin D was used to inhibit *de novo* mRNA synthesis. Knockdown of CPEB4 accelerated p21 mRNA degradation, while overexpression of CPEB4 stabilized p21 mRNA (Figures 5F,H). In addition, CPEB4 knockdown retarded *de novo* synthesis of the p21 protein (Figure 5I). Furthermore, the results of RNA immunoprecipitation assays indicated that CPEB4 is associates with the p21 transcript but not with the GAPDH transcript (Figure 5J). Overall, these findings suggest that CPEB4 binds with the p21 transcript and upregulates p21 expression by increasing p21 mRNA stability and translation.

### CPEB4 Exerts a Tumor-Inhibiting Effect Partially Through Increasing p21 Expression in RCC

**FIGURE 6:**
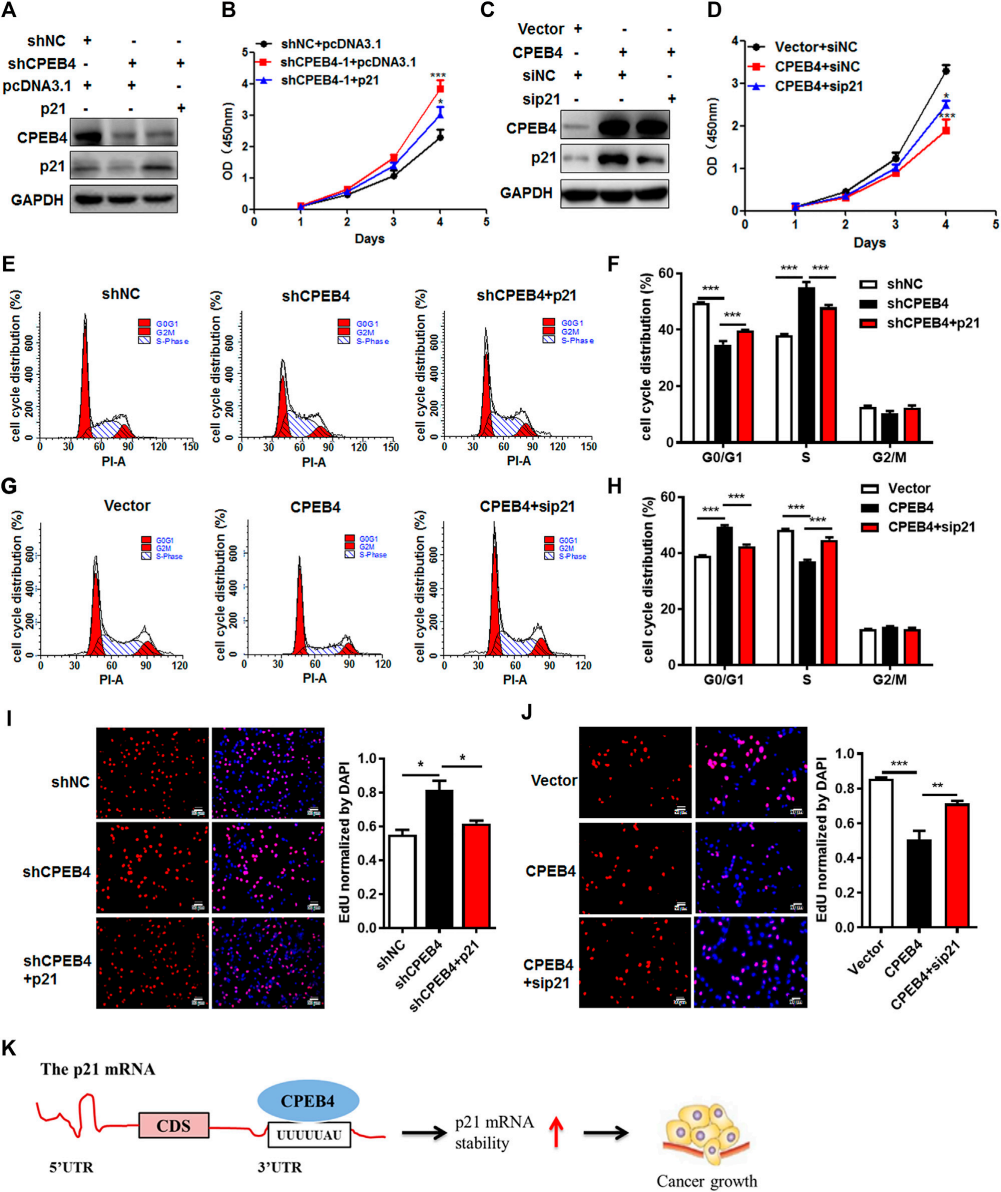
CPEB4 exerts a tumor-inhibiting effect partially through increasing p21 expression in RCC. **(A)** p21 overexpression plasmids were transfected into CPEB4 knockdown ACHN cells. p21 protein expression was detected by western blot. **(B)** p21 overexpression abrogated the pro-proliferative effect of CPEB4 knockdown by CCK-8 assay. **(C)** p21 siRNA were transfected into CPEB4 overexpression 786O cells. p21 protein expression was detected by western blot. **(D)** Silencing of p21 reversed RCC cell proliferation inhibition of CPEB4 overexpression by CCK-8 assay. **(E,F)** p21 overexpression abrogated the G1/S phase transition effect of CPEB4 knockdown by flow cytometry assay. **(G,H)** Silencing of p21 reversed the G1 cell cycle arrest effect of CPEB4 overexpression by flow cytometry assay. **(I,J)** Indicated cells were stained with EDU and DAPI. The red colour indicates EDU-positive nuclei. The statistical analysis of EDU staining was performed by Image-Pro Plus 6.0 software. The dataset is representative example of triplicate experiments. Column graph was mean ± SD of three independent experiments. **(K)** A schematic diagram of the function of CPEB4 in RCC.

To validate the involvement of p21 in the CPEB4-mediated tumor-inhibiting effect, we introduced stable p21 expression into CPEB4-knockdown cells or control cells with an empty vector (Figure 6A). Forced expression of p21 abrogated the pro-proliferative effect of CPEB4 knockdown, which was demonstrated by accelerated cell proliferation (Figure 6B) and G1/S phase transition (Figures 6E,F). In accordance with this result, silencing of p21 reversed RCC cell proliferation inhibition (Figures 6C,D) and G1 cell cycle arrest (Figures 6G,H) caused by CPEB4 overexpression. Furthermore, the results of EDU staining assays also verified the involvement of p21 in the CPEB4-mediated cell proliferation inhibition (Figures 6I,J). Taken together, these findings clearly demonstrate that CPEB4 modulates RCC cell proliferation by inhibiting cell cycle progression partially through increasing p21 expression.

## Discussion

CPEB4 is abnormaly expressed in a variety of tumor cells (Ortiz-Zapater et al., 2011; Chen et al., 2018). To explore the role of CPEB4 in the occurrence and development of renal cancer, we firstly searched the TCGA database and found that the mRNA level of CPEB4 in renal clear cell carcinoma tissues was lower than that in para-carcinoma tissues, and was positively correlated with the overall survival and disease-free survival rates in RCC patients. Additionally, we confirmed CPEB4 expression was downregulated in RCC cell lines and clinical specimens. Furthermore, we found that CPEB4 inhibits RCC cell proliferation both *in vivo* and *in vitro*. These results suggest that CPEB4 acts as a tumor surpressor in RCC.

Previous studies have reported the dependency on CPEB4 when melanoma cells progress through G1/S cell cycle checkpoints (Perez-Guijarro et al., 2016). We checked whether CPEB4 can regulate cell cycle in RCC and found that CPEB4 could induced G1 cell cycle arrest in RCC cells, indicating that CPEB4 may be an important regulator of cell cycle progression in RCC. We further found that CPEB4 overexpression significanctly upregulated tumor surpressor p21, which suppresses cell cycle progression and plays a crucial role in multiple growth suppressor pathways. We hypothesized that CPEB4 modulates the cell cycle in RCC by regulating p21. As expected, CPEB4 upregulated p21 expression through increasing mRNA stability by binding to p21 transcript. Finally, we proved that the proliferation suppression ability of CPEB4 is partly dependent on regulating p21 and cell cycle progression in RCC. Although p21 acted as a downstream effector in CPEB4-induced growth inhibition, it is worth exploring the more in-depth mechanisms involved in the complex interaction of CPEB4 and p21.

Interestingly, preliminary and ChIP-seq data of other group showed that CEBP4 is a direct p53 target gene (Nikulenkov et al., 2012; Zaccara et al., 2014). Human p53 mRNA contains two consensus CPEs in the 3′UTR (D'Erchia A et al., 1999) which can influence the mRNA stability and protein synthesis of p53 (Rosenstierne et al., 2008). CPEB1 upregulates the polyadenylation-induced translation of p53 mRNA in primary human cells (Burns and Richter, 2008). In addition, our recently published study (Di et al., 2021) showed that CPEB2, as a target of p53, can regulate p53 via a negative feedback mechanism. Therefore, CPEB family proteins may have different functions in regulating p53. In this paper, we found that CPEB4 overexpression or konckdown cannot affect p53 expression, but significantly upregulate p21 expression. It will be of great significance to study the synergistic and antagonistic effects of different CPEB family members in the regulation of p53 pathway.

Previous studies showed that CPEB4 seems to play paradoxical roles in different types of tumors. Elevated CPEB4 expression was shown to be an oncogene in several cancers such as pancreatic cancer, glioma and gastric cancer (Hu et al., 2015; Cao et al., 2018). However, a tumor-suppressor role of CPEB4 was demonstrated in hepatocellular carcinoma and non-small cell lung cancer (Tian et al., 2012; Huang et al., 2015). Our data showed that CPEB4 also acted as a tumor surpressor in RCC. Future studies need to explain the reasons for the contradictory roles of CPEB4 in different cancers. Park et al. demonstrated that different CPEB2 isoforms CPEB2A (-exon4) and CPEB2B (+exon4) have the opposite function in breast cancer metastasis (Johnson et al., 2015; DeLigio et al., 2017). CPEB4 have 5 isoforms (A-E), we specifically overexpressed the longest isoform CPEB4A in our study. Whether the contradictory role of CPEB4 in different cancer is related with the alternative splicing of CPEB4 remains to be further investigated in the future.

## Conclusion

In summary, we have demonstrated that lower level expression of CPEB4 is correlated with reduced overall survival and disease-free survival rates in RCC patients. Moreover, CPEB4 inhibits RCC cell proliferation partially dependent on upregulating p21 and further inducing G1 cell cycle arrest. (Figure 6K). This study thus highlights a mechanism underlying which CPEB4 inhibites RCC growth and provides a new promising predictive biomarker for prognosis in patients with RCC.

## Data Availability

The raw data supporting the conclusions of this article will be made available by the authors, without undue reservation.
