# Supplementary material for: CPEB4 Inhibit Cell Proliferation via Upregulating p21 mRNA Stability in Renal Cell Carcinoma
Source: Front Cell Dev Biol. 2021 Dec 16;9:687253. doi: 10.3389/fcell.2021.687253 (PMC8716440; doi:10.3389/fcell.2021.687253)
Supplement: Supplementary file 1 [file Table1.DOCX]

**Table S1**. Primer and siRNA/shRNA sequences

| **Oligonucleotide** | **Sequence** |
| --- | --- |
| CPEB4 F | 5’- TGAGATCACAGCTAGTTTTCGT-3’ |
| CPEB4 R | 5’- TCAATGCATGCATCAATGAGAG-3’ |
| p21 F | 5’-GTCAGAACCCATGCGGCAGCAAG-3’ |
| p21 R | 5’-CAGGTCCACATGGTCTTCCTCTG-3’ |
| Actin F | 5’-AGAAAATCTGGCACCACACC-3’ |
| Actin R | 5’- CTCCTTAATGTCACGCACGA-3’ |
| control siRNA/shRNA | 5’-TTTTCCGAACGTGTCACGTTT-3’ |
| CPEB4 siRNA/shRNA #1 | 5’-GGATCGAATCTCCAGTGTTTT-3’ |
| CPEB4 siRNA/shRNA #2 | 5’-GCATGGAGAGATAGATAAATT-3’ |
