# Supplementary figures and images for: CPEB4 Inhibit Cell Proliferation via Upregulating p21 mRNA Stability in Renal Cell Carcinoma
Source: Front Cell Dev Biol. 2021 Dec 16;9:687253. doi: 10.3389/fcell.2021.687253 (PMC8716440; doi:10.3389/fcell.2021.687253)

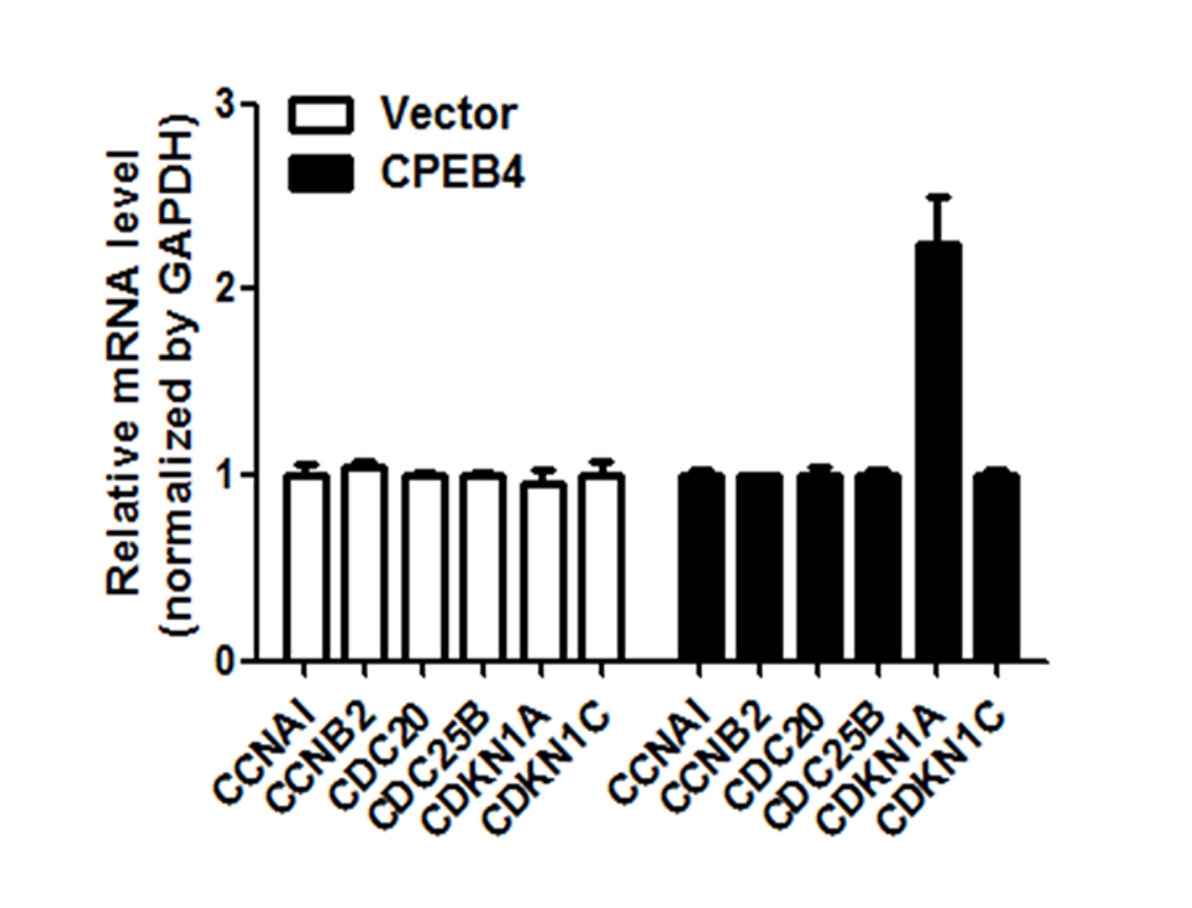

Supplement: Supplementary file 2 [file Image2.TIF]

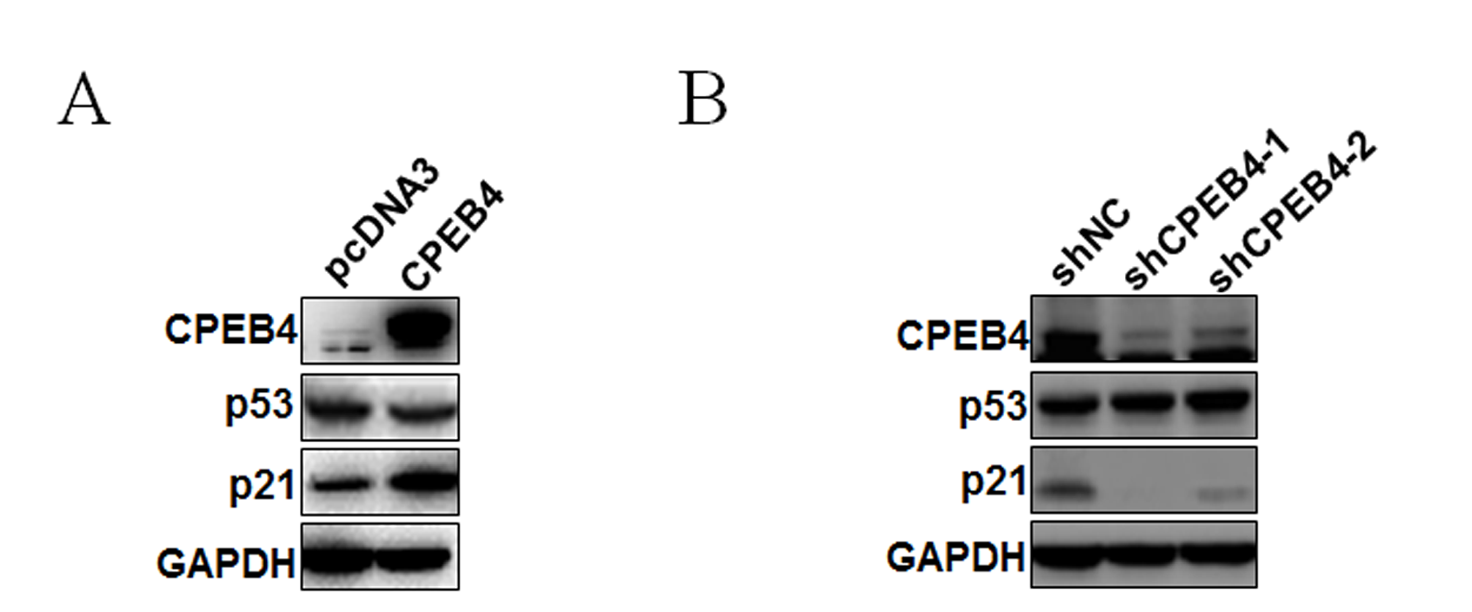

Supplement: Supplementary file 3 [file Image1.TIF]
